# Interaction Profiles of Central Nervous System Active Drugs at Human Organic Cation Transporters 1–3 and Human Plasma Membrane Monoamine Transporter

**DOI:** 10.3390/ijms222312995

**Published:** 2021-11-30

**Authors:** Thomas J. F. Angenoorth, Stevan Stankovic, Marco Niello, Marion Holy, Simon D. Brandt, Harald H. Sitte, Julian Maier

**Affiliations:** 1 Center for Physiology and Pharmacology, Institute of Pharmacology, Medical University of Vienna, Währingerstraße 13A, 1090 Vienna, Austria; thomas.angenoorth@gmx.net (T.J.F.A.); stevan-stankovic@hotmail.com (S.S.); marco.niello@meduniwien.ac.at (M.N.); marion.holy@meduniwien.ac.at (M.H.); julian.maier@meduniwien.ac.at (J.M.); 2 School of Pharmacy and Biomolecular Sciences, Liverpool John Moores University, Byrom Street, Liverpool L3 3AF, UK; s.brandt@ljmu.ac.uk

**Keywords:** ketamine, psilocybin, bupropion, escitalopram, diazepam, tramadol, *O*-desmethyltramadol, cocaine, d-amphetamine, modafinil

## Abstract

Many psychoactive compounds have been shown to primarily interact with high-affinity and low-capacity solute carrier 6 (SLC6) monoamine transporters for norepinephrine (NET; norepinephrine transporter), dopamine (DAT; dopamine transporter) and serotonin (SERT; serotonin transporter). Previous studies indicate an overlap between the inhibitory capacities of substances at SLC6 and SLC22 human organic cation transporters (SLC22A1–3; hOCT1–3) and the human plasma membrane monoamine transporter (SLC29A4; hPMAT), which can be classified as high-capacity, low-affinity monoamine transporters. However, interactions between central nervous system active substances, the OCTs, and the functionally-related PMAT have largely been understudied. Herein, we report data from 17 psychoactive substances interacting with the SLC6 monoamine transporters, concerning their potential to interact with the human OCT isoforms and hPMAT by utilizing radiotracer-based in vitro uptake inhibition assays at stably expressing human embryonic kidney 293 cells (HEK293) cells. Many compounds inhibit substrate uptake by hOCT1 and hOCT2 in the low micromolar range, whereas only a few substances interact with hOCT3 and hPMAT. Interestingly, methylphenidate and ketamine selectively interact with hOCT1 or hOCT2, respectively. Additionally, 3,4-methylenedioxymethamphetamine (MDMA) is a potent inhibitor of hOCT1 and 2 and hPMAT. Enantiospecific differences of R- and S-α-pyrrolidinovalerophenone (R- and S-α-PVP) and R- and S-citalopram and the effects of aromatic substituents are explored. Our results highlight the significance of investigating drug interactions with hOCTs and hPMAT, due to their role in regulating monoamine concentrations and xenobiotic clearance.

## 1. Introduction

Organic cation transporter subtypes 1-3 (OCT1–3; SLC22A1–3, respectively; OCTs) and the plasma membrane monoamine transporter (PMAT; SLC29A4) are poly-specific facilitative transporters which are involved in the uptake and elimination of various endogenous compounds, most notably monoamines, as well as of drugs, xenobiotics, and toxins [1]. In addition to the high-affinity, low-capacity solute carrier 6 (SLC6) neurotransmitter-sodium symporters of norepinephrine (norepinephrine transporter; NET; SLC6A2), dopamine (dopamine transporter; DAT; SLC6A3) and serotonin (serotonin transporter; SERT; SLC6A4), the low-affinity, high-capacity OCTs as well as PMAT are of paramount importance for the maintenance of monoaminergic equilibrium in the brain [2,3]. In addition, OCTs contribute to protection against noxious compounds by intestinal absorption as well as hepatic and renal excretion [4,5,6]. Indeed, hOCT1 and hOCT2 transport numerous compounds in the small intestine, liver and kidneys, with OCT1 being primarily expressed in the liver and OCT2 predominantly acting in the kidney, while both are, together with hOCT3 and hPMAT, also found in the brain, albeit at a lower level [7]. By contrast, hOCT3 and hPMAT were found to be highly expressed in the brain, alongside their less ubiquitous occurrence in peripheral organs, such as the heart for hOCT3 [8]. Here, along with hOCT1 and 2, they participate in the regulation of the monoaminergic equilibrium, and their dysfunction has been associated with disturbance of monoaminergic pathways, leading to various psychiatric disorders and neurodegenerative diseases [9,10,11,12,13].

Due to their involvement in the excretion of drugs and metabolites, members of the OCT family have overlapping substrate and inhibitor profiles [5,14]. Substrates are classically transported into the cells by the transporter, while inhibitors bind to the transporter in the outward-facing confirmation and prevent uptake [15]. Identification of compounds interacting with OCTs and PMAT furthers knowledge of targets and effects in the central nervous system (CNS) and, additionally, allows for the explanation of substances’ observed pharmacokinetic properties. Given the plethora of CNS active substances that are well-known for their interplay with SLC6 neurotransmitter-sodium symporters (summarized as monoamine transporters; MATs), there is also a growing amount of interest in their interaction with the human OCTs as well as PMAT, since they are less well-researched potential targets in the monoamingergic system [16,17,18,19,20,21]. We have recently reported that several psychoactive substances, classified primarily as inhibitors or substrates of the MATs, equipotently interact with certain OCTs [2,22].

To further extend these observations, we screened 17 substances with CNS activity that were categorized as follows: antidepressants (bupropion, S-citalopram, R-citalopram), antiepileptics (diazepam, phenobarbital), psychostimulants (cocaine, *d*-amphetamine, R- and S-α-pyrrolidinovalerophenone (R- and S-α-PVP)), emerging therapeutics (3,4-methylenedioxymethamphetamine (MDMA), psilocin, ketamine), the ketamine derivative 2-fluoro-deschloroketamine, drugs for attention deficit hyperactivity disorder (ADHD) and narcolepsy (methylphenidate, modafinil) and analgesics (tramadol and its metabolite *O*-desmethyl-tramadol). 2-fluoro-deschloroketamine and *O*-desmethyltramadol can be classified as new psychoactive substances that are not yet controlled by legislation [23]. Considering the myriad of already established interaction profiles on SLC6 transporters, we tried to start an analogous process for the low-affinity, high-capacity monoamine transporters, as doing so will help to further elucidate the pharmacological interaction of compounds with OCTs and PMAT. This approach will offer more information on these transporters’ rich pharmacology and potentially lead to clinical implications for patients with OCT polymorphisms impacting substrate translocation [24]. In addition, the discovery of previously unreported interactions of well-known psychoactive substances with OCTs and PMAT might inspire the usage of compounds as potential scaffolds to develop new clinically useful drugs.

## 2. Results

**Figure 1:**
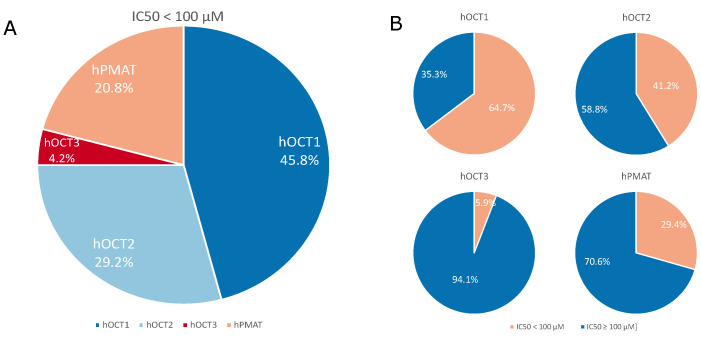
Graphical representation of the percentage of screened compounds inhibiting the substrate uptake of the transporters of interest with an half-maximal inhibitory concentration ($IC_{50}$) below 100 μM. **A**: 45.8% of compounds inhibit hOCT1 (dark blue), 29.2% inhibit hOCT2 (light blue), 20.8% hPMAT (orange) and 4.2% inhibit hOCT3 (red). **B**: 11 out of 17 compounds inhibit hOCT1, 7 compounds inhibit hOCT2, 1 compound inhibit hOCT3 and 5 compounds inhibit hPMAT.

Uptake inhibition assays were performed on human embryonic kidney 293 (HEK293) cells stably expressing hOCT1, hOCT2, hOCT3 or hPMAT to assess the compounds’ potency to inhibit the uptake of 1-methyl-4-phenylpyridinium ([${}^{3}$H]-MPP${}^{+}$). We found several substances to be inhibitors of substrate uptake by hOCT1, and fewer interacted with hOCT2 and hPMAT, whereas interactions with hOCT3 were exceedingly rare (see Figure 1). In summary, only 1 compound (∼6%) inhibited hOCT3, 5 compounds (∼29%) inhibited hPMAT, 7 compounds (∼41%) inhibited hOCT2, and 11 compounds (∼65%) inhibited hOCT1 with an half-maximal inhibitory concentration ($IC_{50}$) value lower than 100 μM (see Figure 1B).

**Figure 2:**
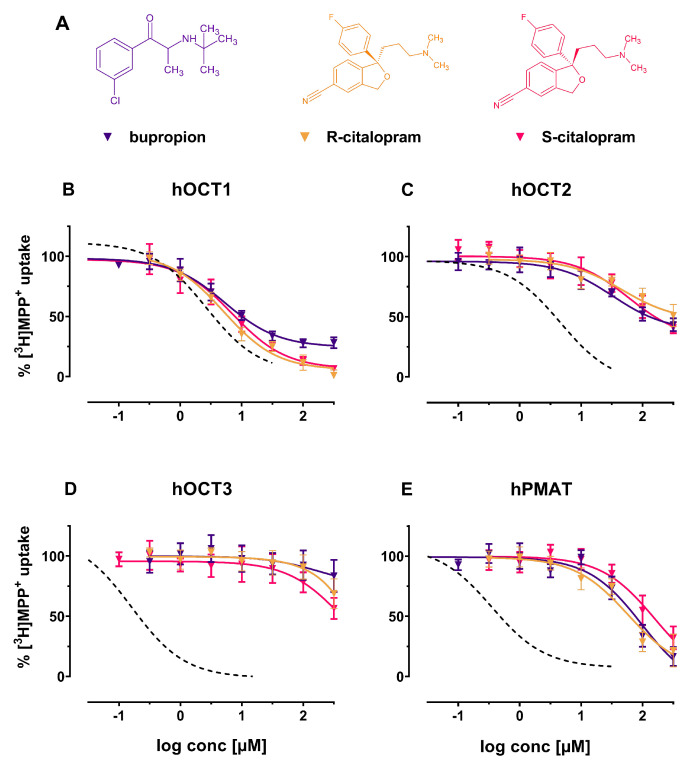
(**A**) Chemical structures of the herein investigated antidepressant compounds. From left to right: bupropion (violet), R-citalopram (yellow) and S-citalopram (red); Effects of the above-mentioned compounds (including decynium-22; dashed line) on (**B**) hOCT1, (**C**) hOCT2, (**D**) hOCT3 and (**E**) hPMAT of uptake of tritiated MPP${}^{+}$ in HEK293 cells stably expressing the respective transporter.

The screened antidepressants had close to identical interaction profiles on all transporters (see Figure 2) with only hOCT1 uptake being inhibited at pharmacologically relevant concentrations, with $IC_{50}$ values between 5.11 μM (95%-confidence interval (CI): 4.11–6.37) to 7.15 μM (95%-CI: 5.59–9.15) (Table 1). Interestingly, R-citalopram, S-citalopram and bupropione did not interact as potently with hOCT2 and hOCT3 (see Figure 2C,D). The examined antidepressants inhibited hPMAT with low potency at high micromolar concentrations (Figure 2E).

**Figure 3:**
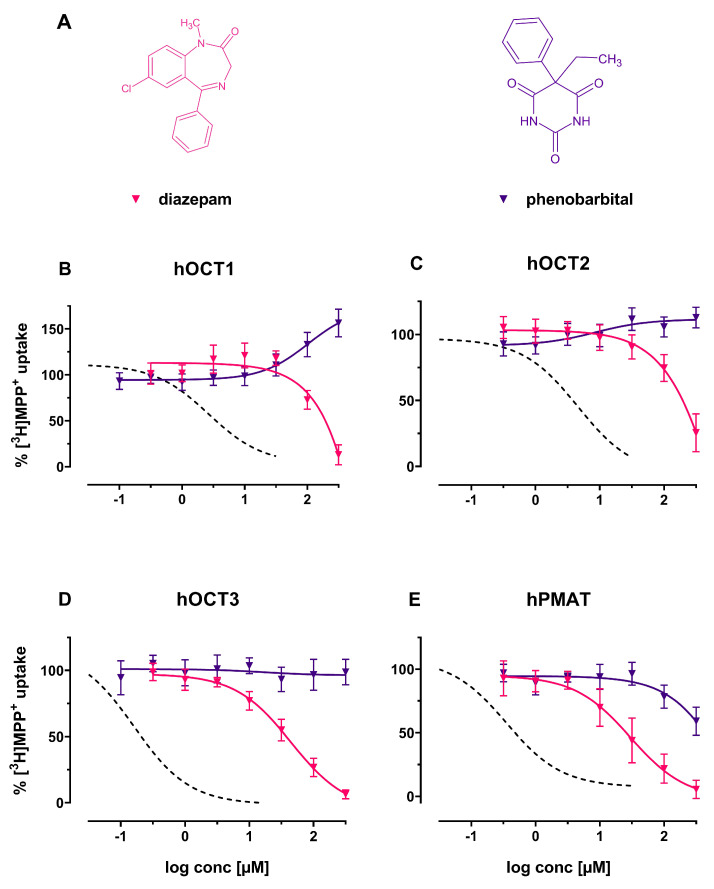
(**A**) Chemical structures of the herein investigated antiepileptics diazepam (red) and phenobarbital (violet); (**B**–**E**): Effects of the above-mentioned compounds (including decynium-22; dashed line) on (**B**) hOCT1, (**C**) hOCT2, (**D**) hOCT3 and (**E**) hPMAT of uptake of tritiated MPP${}^{+}$ in HEK293 cells stably expressing the respective transporter.

Diazepam fully inhibited substrate uptake of all transporters (see Figure 3B–E), reaching half-maximal inhibition at 44.46 μM (95%–CI: 36.04–54.85) and 29.81 μM (95%-CI: 18.41–48.27) at hOCT3 and hPMAT, respectively, while weakly interacting with hOCT1 and hOCT2 ($IC_{50}$>100μM). Phenobarbital treatment induced increased uptake of substrate at increasing concentrations at hOCT1 and hOCT2, while not interacting with the other transporters at pharmacologically relevant concentrations.

**Figure 4:**
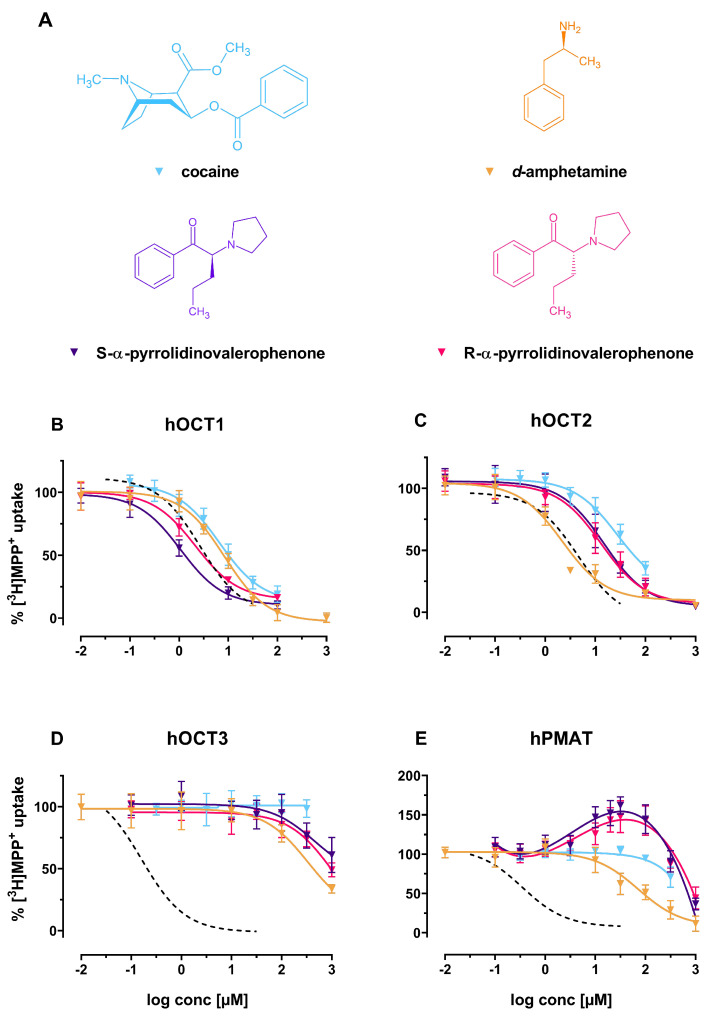
(**A**) Chemical structures of the herein investigated psychostimulants. From left to right: cocaine (blue), *d*-amphetamine (yellow), S-α-pyrrolidinovalerophenone (violet) and R-α-pyrrolidinovalerophenone (red); (**B**–**E**): Effects of the above-mentioned compounds (including decynium-22; dashed line) on (**B**) hOCT1, (**C**) hOCT2, (**D**) hOCT3 and (**E**) hPMAT of uptake of tritiated MPP${}^{+}$ in HEK293 cells stably expressing the respective transporter.

The psychostimulants *d*-amphetamine, cocaine, R- and S-α-PVP showed similar inhibition profiles at hOCT1 and hOCT2 (Figure 4B,C) with $IC_{50}$s ranging from 1.07 (95%-CI: 0.81–1.40) to 15.02 μM (95%-CI: 11.28–20.00), with the exception of an $IC_{50}$ of 27.80 μM (95%-CI: 19.17–40.32) of cocaine at hOCT2 (see Figure 4C). Furthermore, no pharmacologically relevant interactions with hOCT3 and hPMAT could be detected (Figure 4D,E). R- and S-α-PVP treatment lead to increased uptake of substrate at hPMAT (Figure 4E).

**Figure 5:**
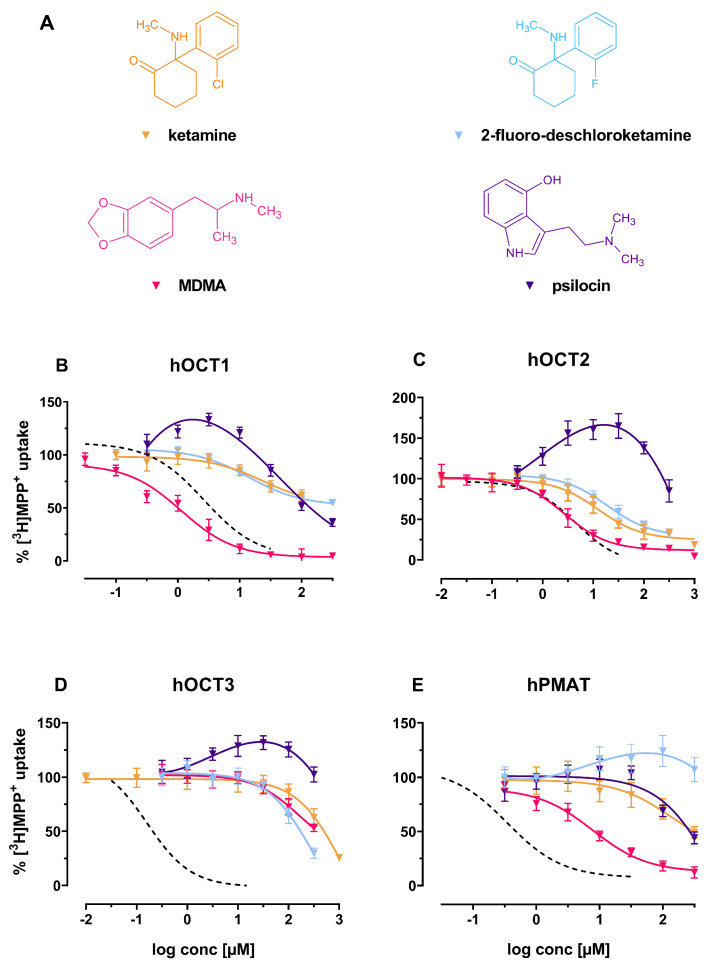
(**A**) Chemical structures of the herein investigated new therapeutics. From left to right: ketamine (yellow), 2-fluoro-deschloroketamine (blue; non-therapeutic derivative of ketamine), MDMA (red) and psilocin (violet): (**B**–**E**): Effects of the above-mentioned compounds (including decynium-22; dashed line) on (**B**) hOCT1, (**C**) hOCT2, (**D**) hOCT3 and (**E**) hPMAT of uptake of tritiated MPP${}^{+}$ in HEK293 cells stably expressing the respective transporter.

While ketamine selectively and potently inhibited substrate uptake of hOCT2, its derivative 2-fluoro-des-chloroketamine is less potent, but similarly selective $(IC_{50}$ of 12.46 (95%-CI: 9.71–15.98) and 19.18 μM (95%-CI: 14.89–24.70), respectively). Both compounds did not fully inhibit the other transporters at pharmacologically relevant concentrations. Psilocin, the active metabolite of psilocybin, did not inhibit any of the investigated transporters, but rather caused an increase in substrate uptake in the low micromolar range at the OCTs (see Figure 5B–D). 3,4-Methylenedioxymethamphetamine (MDMA) has high substrate uptake inhibiting potencies at hOCT1, 2 ($IC_{50}$ of 1.14 (95%-CI: 0.90–1.44) and 2.71 μM (95%-CI: 2.22–3.31) respectively) and hPMAT ($IC_{50}$ of 7.77 μM; 95%-CI: 5.92–10.21), while not inhibiting uptake by hOCT3.

**Figure 6:**
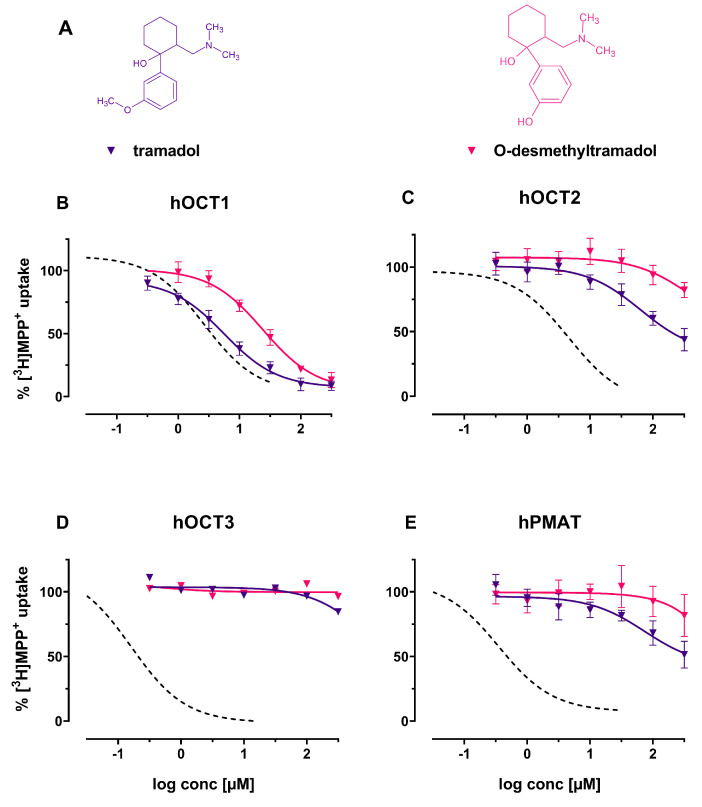
(**A**) Chemical structures of the herein investigated analgesics tramadol (violet) and *O*-desmethyltramadol (red); (**B**–**E**): Effects of the above-mentioned compounds (including decynium-22; dashed line) on (**B**) hOCT1, (**C**) hOCT2, (**D**) hOCT3 and (**E**) hPMAT of uptake of tritiated MPP${}^{+}$ in HEK293 cells stably expressing the respective transporter.

Tramadol and its active metabolite *O*-desmethyltramadol both inhibit hOCT1 substrate uptake at low micromolar concentrations ($IC_{50}$ of 5.60 (95%-CI: 4.65–6.75) and 24.16 μM (95%-CI: 19.12–30.54) respectively) (see Figure 6B). Tramadol also interacts with hOCT2 and hPMAT, but fails to fully inhibit the transporters (Figure 6C,E). No interaction of both tramadol and *O*-desmethylotramadol with hOCT3 was observed (Figure 6D).

**Figure 7:**
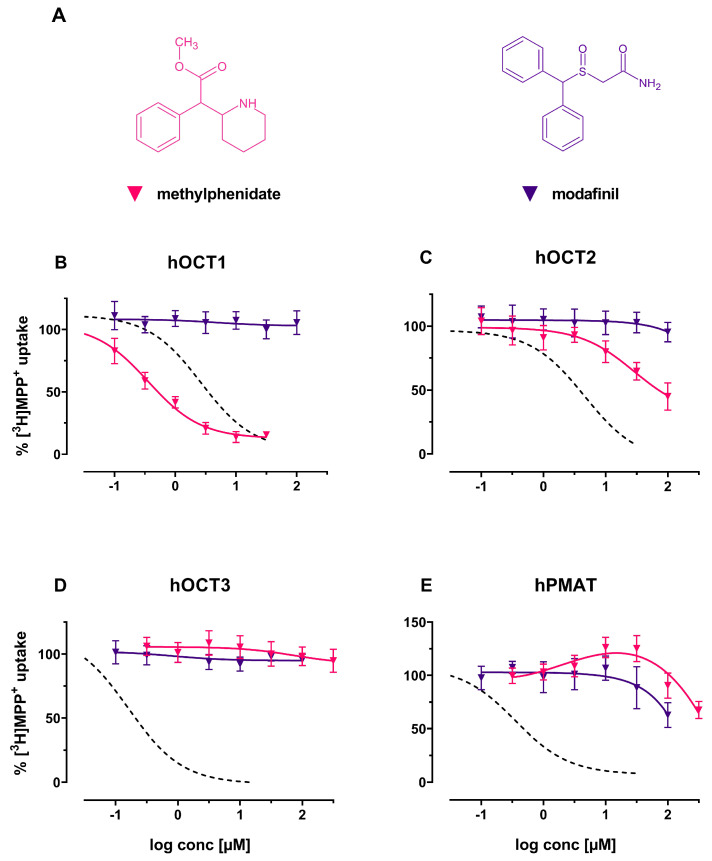
(**A**) Chemical structures of the herein investigated drugs to treat narcolepsy modafinil (violet) and ADHD methylphenidate (red); (**B**): Effects of the above-mentioned compounds (including decynium-22; dashed line) on (**B**) hOCT1, (**C**) hOCT2, (**D**) hOCT3 and (**E**) hPMAT of uptake of tritiated MPP${}^{+}$ in HEK293 cells stably expressing the respective transporter.

As seen in Figure 7B–E, modafinil did not interact with hOCTs and hPMAT at pharmacologically relevant concentrations. In contrast, methylphenidate potently interacts with hOCT1 (Figure 7B) ($IC_{50}$ of 0.36 μM; 95%-CI: 0.27–0.46), while not fully blocking any other investigated transporter (see Figure 7C–E).

## 3. Discussion

To date, many substances have been investigated concerning their interaction with the high-affinity, low-capacity SLC6 MATs due to their long history of use as clinically relevant drug targets, in addition to their well-established role in regulating the clearance of monoamines from the synaptic cleft and maintaining monoaminergic equilibrium in the CNS. However, a growing number of studies shows that the low-affinity, high-capacity organic cation transporters (OCT1–3), as well as the plasma membrane monoamine transporter (PMAT), are also distinctly involved in monoamine reuptake from the synaptic cleft [11,25]. Furthermore, they transport and eliminate xenobiotics in the periphery, lending them pharmacological and clinical relevance [11,26,27,28,29,30,31].

Herein, we intended to systematically determine the interaction profiles of 17 psychoactive substances, including medical drugs, which have mostly been shown to interact with MATs, on hOCT1–3 and hPMAT [32,33,34,35,36]. The presented data thus fill a veritable research gap, since only a few of the compounds have previously been investigated concerning their interaction at the OCTs and PMAT (see Supplementary Table S1).

The first striking finding is that the various chemical classes of psychoactive compounds investigated herein predominantly interact with hOCT1, and less pronouncedly with hOCT2 and hPMAT. Previous studies have shown many medically relevant compounds, such as metformin, to interact with hOCT1, in turn influencing their pharmacokinetic fate [37]. Furthermore, polymorphisms of hOCT1 have been shown to influence substrate translocation and drug–drug interactions [38]. Overall, OCT1 polymorphisms seem to have potential clinical consequences, but further research needs to be undertaken [31]. In addition, FDA (Food and Drug Administration) and EMA (European Medicines Agency) recommend investigations of potential interactions with hOCT2 for drugs which are primarily renally excreted [39,40]. Thus, for compounds potently interacting with hOCTs and hPMAT, the occurrence of polymorphisms in patients, differently affecting pharmacodynamic and -kinetic fates and potentially the occurrence and severity of side-effects, must be considered in future investigations and substantiated clinically.

Currently, only few selective hOCT or hPMAT inhibitors are known, which can be explained by the high degree of sequence homology between transporters [25]. In particular, hOCT1 and 2 share approximately 70% amino acid sequence identity [2,14]. It is therefore not surprising to see an overlap of compounds interacting with hOCT1 and 2. Still, we located distinct differences between these two transporters regarding some of the compounds tested. Bupropion selectively interacts with hOCT1. This contradicts results reported in previous publications [16]. Diverging data between our results and those gathered from earlier studies might be explained by differences in experimental and laboratory conditions, as well as the established cell lines across studies [41]. In addition, tramadol, its metabolite *O*-desmethyltramadol, and both citalopram enantiomers were selective hOCT1 inhibitors. Strikingly, we found methylphenidate to potently and selectively interact with hOCT1, even more potent than the unspecific OCT and PMAT inhibitor decynium-22 [1]. Furthermore, ketamine potently interacts with hOCT2, while showing little activity at other hOCTs and hPMAT. Amphoux et al. (2006) reported similar results for the OCTs but did not investigate hPMAT [26]. This highlights the interesting finding that some compounds explicitly differ in their interactions with hOCT1 and hOCT2, despite the high degree of sequence homology between the two transporters. One possible explanation for this discrepancy could be differences between substrate binding sites. Amino acid residues that are critical for substrate specificity, which are localized in regions formed by transmembrane helices crucial for substrate binding, were found to differ between OCT1 and OCT2, and might explain variations in compound interactions with those transporters [42,43]. Still, the exact molecular basis for preference of compounds for hOCT1 or 2 has not been clearly established yet [2,14,44]. While we could not solve this gap in knowledge, our investigation led to the discovery of scaffolds that can be used for the development of selective inhibitors of OCTs, expanding pharmacological and structural understanding of this transporter family.

hOCT3 shares less sequence identity (50%) with the other OCTs and it has been previously shown that many unselective inhibitors and substrates of the organic cation transporters are least efficacious at hOCT3 [14]. In our study, diazepam not only proved to be a clearly more potent inhibitor of substrate uptake at hOCT3 when compared to hOCT1 and 2, but additionally, was the only herein tested substance to exhibit any pharmacologically relevant effect on substrate uptake by hOCT3. A previous study reported an $IC_{50}$ of 2 μM for diazepam at hOCT3, which is much lower than the one we have measured ($IC_{50}$ of 44.46 μM) [18]. The discrepancy may be explained by the different tracer used, with Massmann and colleagues having employed fluorescent 4-(4-(dimethylamino)styryl)-N-methylpyridinium iodide (ASP${}^{+}$), while we performed uptake inhibition assays with radioactively labelled MPP${}^{+}$ [41]. In addition, diazepam relatively potently interacted with hPMAT, which was previously unreported.

In an investigation of α-pyrrolidinopropiophenone derivatives, we have previously observed that differences in aromatic ring substituents affected the hOCTs and hPMAT differently [22]. Differences in interaction with hOCT1 and 2 were marginal compared to their impact on hOCT3 and hPMAT. Here, we examined ketamine and its derivative 2-fluoro-deschloroketamine, again noticing only small differences caused by different aromatic ring substituents, although the potency to inhibit hOCT2 substrate uptake decreased for the derivative. This lack of sensitivity towards substituents of the aromatic ring differentiates hOCT1 and 2 from SLC6 MATs where, for example in the case of methcathinone analogs, fluorination of the aromatic ring improved SERT selectivity over DAT [35,45,46]. On the other hand, structural differences between tramadol and its main metabolite *O*-desmethyltramadol led to relevant changes in hOCT1 affinity, as tramadol inhibited substrate uptake of the transporter with a more than four-fold higher potency ($IC_{50}$ of 5.60 μM) than *O*-desmethyltramadol ($IC_{50}$ of 24.16 μM). Future studies need to focus on identified, potent chemical scaffolds and systematically investigate effects of substituents in larger-scale structure–activity relationship investigations.

To our knowledge, no previous studies have analyzed the effects of these compounds at human OCTs or hPMAT. Thus, we examined pharmacodynamic properties of S-citalopram and R-citalopram, as well as S- and R-α-PVP. Generally, we saw no distinct enantioselective differences in uptake inhibition between the two compounds on any transporter, emphasizing the robustness of OCTs and PMAT concerning drug–transporter interactions, which discerns them from MATs [23,47,48].

We observed the surprising phenomenon that treatment with higher concentrations of some compounds, most markedly phenobarbital, psilocin and α-PVP, led to elevated MPP${}^{+}$ uptake at hOCT1, 2 and hPMAT. Consistent with this finding is a previous study of Ahlin and colleagues, who reported increased uptake after cell treatment with high concentrations of phenobarbital [49]. One possible explanation for this striking phenomenon might include allosteric effects on the orthosteric site of the transporter. Consequently, an allosteric ligand would change the conformational dynamics of the transporter protein and thereby regulate interaction between substrates and ligand binding site [50]. In line with this phenomenon, an earlier study detailed the existence of a high-affinity binding site at OCTs, proposing it to partake in the effectivity of MPP${}^{+}$ uptake through positively affecting the transport executed by two low-affinity transporting sites [51]. However, further studies are needed to unveil the exact molecular mechanisms.

Traditionally, decyinum-22 was oftentimes used as a positive control due to its high inhibitory potency at all hOCTs and hPMAT. In the present investigation, we show multiple compounds to have similar or even lower $IC_{50}$ values. *d*-Amphetamine proved to be a highly potent hOCT1 and 2 substrate uptake inhibitor with $IC_{50}$ values in the low micromolar range, while not interacting with hOCT3 at concentrations lower than 100 μM, which is consistent with a previous study by Amphoux et al. (2006) [26]. Furthermore, in our experiments, MDMA was potently interacting with hOCT1 and 2, an aspect which is to some extent consistent with a previous study showing fairly low Ki values [26]. In addition, the compound potently interacted with hPMAT, an interaction which had not been previously described. The most striking finding is that methylphenidate potently and selectively inhibits substrate uptake at hOCT1 in the nanomolar range. Due to the facts that (i) similar values have been reported as $IC_{50}$ values at the primary targets of methylphenidate, hDAT and hNET [52], and (ii) the interindividual variability concerning appropriate dosing and avoidance of toxicity is high [53], it is possible that hOCT1 polymorphisms, which have been reported to frequently occur in the general population [20], might affect the pharmacokinetic and pharmacodynamic fate of methylphenidate in vivo in a clinically relevant manner, warranting further investigation in this particular case but also for other compounds potently interacting with hOCT1 and hOCT2 [31].

## 4. Materials and Methods

### 4.1. Chemicals and reagents

The compounds of interest were obtained from either Sigma-Aldrich (St. Louis, MO, USA) or LGC Standards (Teddington, UK). S- and R-α-PVP was graciously provided by the NIDA Drug Supply Program. 2-fluoro-deschloroketamine was kindly provided by S. Brandt. All other chemicals and cell culture supplies were obtained from Sigma-Aldrich (St. Louis, MO, USA) and Sarstedt (Nuembrecht, Germany).

### 4.2. Cell Culture

HEK293 cells were transfected with a plasmid, encoding the transporter of interest and an N-terminal YFP-tag, carried out using jetPRIME${}^{©}$ (Polyplus Transfection; (VWR International GmbH, Vienna, Austria)) reagent (for a 10 cm dish with 1–2 ×$10^{6}$ cells in 10 mL serum containing medium at 60–80% confluency: 500 μL jetPRIME${}^{©}$ buffer, 10 μg DNA and 20 μL jetPRIME${}^{©}$ reagent) and selection pressure maintained for two weeks [2,54,55]. Subsequently, 500,000 cells were FACS-sorted (fluorescence-activated cell sorting) according to expression level to establish cell lines stably expressing the protein of interest. Fluorescence microscopy images of cells expressing respective YFP-tagged transporters are seen in Supplementary Figure S1. Concentration dependent uptake of tritiated substrate by transiently transfected cell lines expressing hOCT1–3 and hPMAT is shown in Supplementary Figure S2. For uptake inhibition assays, cells were cultured at a subconfluent (80–90% density) state in high-glucose Dulbecco’s Modified Eagle Medium (DMEM ), enriched with 10% heat-inactivated Fetal Bovine Serum (FBS) and penicillin/streptomycin (PS; 100 U × 100 mL${}^{-1}$, each). Selection pressure was maintained by the addition of geneticin (50 mg × mL${}^{-1}$) at 37 ${}^{\circ }$C and 5% CO${}_{2}$ in a humidified incubator. At a density of 60,000 cells per well, HEK293 cells expressing the respective transporter were seeded onto poly-D-lysine-coated wells 24 h prior to uptake inhibition experiments in a final volume of 200 μL per well.

### 4.3. Uptake Inhibition Assays

**Figure 8:**
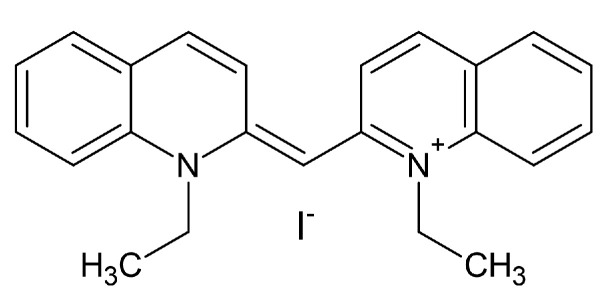
Chemical structure of decynium-22 (D22). D22 was used in all subsequent graphs as a highly potent transporter inhibitor for the assessment of unspecific uptake of the tritiated substrate ${[}^{3}H]$-MPP${}^{+}$.

Prior to the addition of the respective compounds, DMEM was removed from all wells and replaced with 200 μL of Krebs-HEPES-buffer (KHB; 10 mM HEPES, 120 mM NaCl, 3 mM KCl, 2 mM CaCl${}_{2}$, 2 mM MgSO${}_{4}$ and 20 mM D-glucose, pH adjusted to 7.3) at room temperature. Cells were exposed to a pre-incubation solution containing the compound of interest in the respective concentration (dissolved in Krebs-HEPES-buffer) for 10 min with a total volume of 50 μL, which was then replaced with a 50 μL uptake solution containing 0.05 μM of tritiated 1-methyl-4-phenylpyridinium ([${}^{3}$H]MPP${}^{+}$) as substrate, and the compound of interest in respective concentrations, for 10 minutes each. Finally, uptake was terminated by washing with 200 μL of ice-cold (4 ${}^{\circ }$C) KHB, after which cells were lysed with 200 μL of 1% sodium dodecyl sulphate (SDS). After transferring the solution of each well into 6 mL counting vials containing 2 mL of scintillation cocktail, the vials were measured with a beta-scintillation counter for quantification of the uptake of tritiated substrate. Decynium-22 (D22) shows robust inhibitions at low micromolar concentrations at all hOCTs and at hPMAT ranging between 0.16 μM at hOCT3 and 4.56 μM at hOCT2. D22 is depicted as a reference inhibition curve (dotted line) in all Figure 2, Figure 3, Figure 4, Figure 5, Figure 6 and Figure 7. The structural formula can be seen in Figure 8. To obtain specific data, non-specific uptake was assessed in the presence of 100 μM D22 and subtracted from the total data. The 100% values were defined as uptake in the absence of a tested substance, defining maximal uptake capacity. Inhibitions of the compound of interest were described as a percentage of the maximal uptake capacity. While uptake inhibition assays are an effective tool for investigating the interaction of compounds with transporters of interest, they are not able to distinguish substrates from inhibitors, per se. Thus, we cannot rule out that some of the investigated compounds are substrates rather than inhibitors. Future studies are needed to investigate this uncertainty.

### 4.4. Data and Statistical Analysis

Half-maximal inhibitory concentration ($IC_{50}$) values of each substance were calculated and plotted using GraphPad Prism 8.4.3 (GraphPad Software Inc., San Diego, CA, USA). $IC_{50}$ was determined by non-linear regression, solving the equation $Y=Bottom+\left(Top-Bottom\right)/\left(1+10^{X-LogEC_{50}}\right)$. Data which displayed an increased uptake at higher concentrations was fit as a third-order polynomial, solving the equation: $Y=B0+B1*X+B2*X^{2}+B3*X^{3}$. All data stem from a minimum of three separate experiments (*n*≥ 3) executed in triplicates and are shown as mean ± SD. All colors were chosen to be color-blind-friendly using a color brewer [56].

## 5. Conclusions

The investigation of the inhibitory potential of substances acting on the central nervous system has revealed some of them to inhibit the substrate uptake of human low-affinity organic cation transporters (OCT1–3) and PMAT in sub-micromolar or a low micromolar concentration. Bupropion and methylphenidate were found to be potent selective inhibitors of substrate uptake at hOCT1, with the latter interacting with hOCT1 more potently than the well-established OCT blocker decynium-22. MDMA was found to interact with hOCT1 and 2 more potently than decynium-22, as was *d*-amphetamine at hOCT2. Ketamine showed interaction selectivity and high uptake inhibitory potential at hOCT2. Further, tramadol, its main metabolite *O*-desmethyltramadol, and both citalopram enantiomers are selective inhibitors of the substrate uptake at hOCT1. Diazepam was the only tested compound to interact with hOCT3. We have found only small differences in the interaction caused by different aromatic ring substituents, as exemplified by ketamine and its derivative, 2-fluoro-deschloroketamine. Notably, we saw no enantioselective effect of S-citalopram and R-citalopram, or S- and R-α-PVP, accentuating the robustness of human OCTs and hPMAT. Considering the growing interest in pharmacological interaction of compounds with human OCTs and PMAT, our study provides important information regarding the complex interaction between a range of CNS-active substances and low-affinity transporters, suggesting some clinically relevant drugs as lead structures for the development of more selective inhibitors of these relatively understudied transporters [2,57].

## Figures and Tables

**Table 1:** Heat map portraying the inhibitory potency (in μM) of screened compounds at hOCT1–3 and hPMAT. Lower values are highlighted in red and higher in shades of blue (see legend).

Substance	Transporters			
	hOCT1	hOCT2	hOCT3	hPMAT
Bupropion	5.36			96.96
S-Citalopram	7.15			
R-Citalopram	5.11			58.32
Diazepam			44.46	29.81
Phenobarbital				
*d*-Amphetamine	8.39	2.21		71.77
Cocaine	6.66	27.80		
R-α-PVP	2.15	13.09		
S-α-PVP	1.07	15.02		
Ketamine		12.46		
2-Fluoro-deschloroketamin		19.18		
MDMA	1.14	2.71		7.77
Psilocin				
Tramadol	5.60			
*O*-Desmethyltramadol	24.16			
Metylphenidate	0.36			
Modafinil				
Decynium-22	2.66	4.56	0.16	0.35

$IC_{50}$[μM] 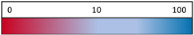

## Data Availability

The data presented in this study are available from the corresponding author upon reasonable request.

## Supplementary Materials

The following are available online at https://www.mdpi.com/article/10.3390/ijms222312995/s1.
