# Supplementary material for: Interaction Profiles of Central Nervous System Active Drugs at Human Organic Cation Transporters 1–3 and Human Plasma Membrane Monoamine Transporter
Source: Int J Mol Sci. 2021 Nov 30;22(23):12995. doi: 10.3390/ijms222312995 (PMC8657792; doi:10.3390/ijms222312995)
Supplement: Supplementary file 1 [file ijms-22-12995-s001.zip › ijms-1451274-supplementary-updated.pdf]

International Journal of *Molecular Sciences*

MDPI

Supplementary Material

Interaction Profiles of Central Nervous System Active Drugs at Human Organic Cation Transporters 1–3 and Human Plasma Membrane Monoamine Transporter

Thomas J. F. Angenoorth [1,†], Stevan Stankovic [1,†], Marco Niello [1], Marion Holy [1], Simon D. Brandt [2], Harald H. Sitte [1,*] and Julian Maier [1]

1 Center for Physiology and Pharmacology, Institute of Pharmacology, Medical University of Vienna, Währingerstraße 13A, 1090 Vienna, Austria; thomas.angenoorth@gmx.net (T.J.F.A.); stevan-stankovic@hotmail.com (S.S.); marco.niello@meduniwien.ac.at (M.N.); marion.holy@meduniwien.ac.at (M.H.); julian.maier@meduniwien.ac.at (J.M.)

2 School of Pharmacy and Biomolecular Sciences, Liverpool John Moores University, Byrom Street, Liverpool L3 3AF, UK; s.brandt@ljmu.ac.uk

* Correspondence: harald.sitte@meduniwien.ac.at

† These authors contributed equally to this work.

check for updates

Citation: Angenoorth, T.J.F.; Stankovic, S.; Niello, M.; Holy, M.; Brandt, S.D.; Sitte, H.H.; Maier, J. Interaction Profiles of Central Nervous System Active Drugs at Human Organic Cation Transporters 1-3 and Human Plasma Membrane Monoamine Transporter. *Int. J. Mol. Sci.* **2021**, *22*, 12995. https://doi.org/10.3390/ijms222312995

Academic Editor: Giuliano Ciarimboli

Received: 21 October 2021
Accepted: 28 November 2021
Published: 30 November 2021

Publisher's Note: MDPI stays neutral with regard to jurisdictional claims in published maps and institutional affiliations.

Copyright: © 2021 by the authors. Licensee MDPI, Basel, Switzerland. This article is an open access article distributed under the terms and conditions of the Creative Commons Attribution (CC BY) license (https://creativecommons.org/licenses/by/4.0/).

Supplementary Materials

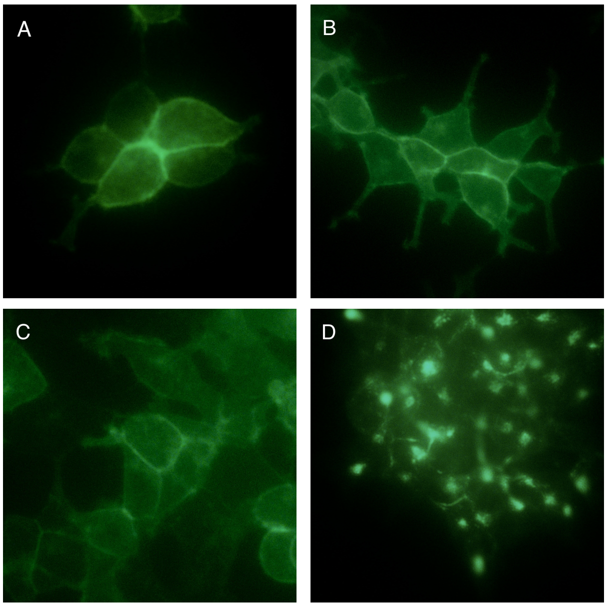

Figure S1. Microscopy images depicting cells expressing YFP-tagged transporters of interest: (**A**): hOCT1, (**B**): hOCT2, (**C**): hOCT3 and (**D**): hPMAT.

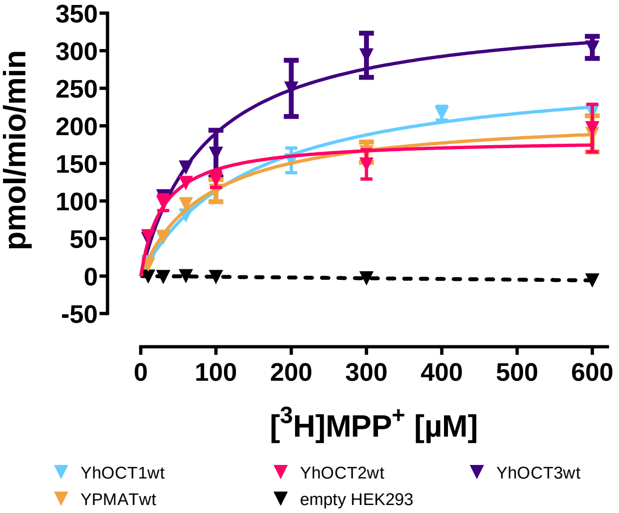

Figure S2. Representative experiments depicting concentration dependant uptake of tritiated MPP$^+$ by the transporters (hOCT1 light blue, hOCT2 red, hOCT3 violet and hPMAT orange, all transiently transfected) of interest and empty HEK293 cells (dashed line) as control.

Table S1. IC_{50} comparison table. Red boxes indicate that no respective data for comparison was found. n.s. = non-significant. Red text color: experiments were performed with ASP$^+$ as substrate. Orange text color: values show the percentage of inhibition at 100 µM of the respective compound. Blue text color: Ki (µM) values were measured.

	Substance	hOCT1		hOCT2		hOCT3		hPMAT	
		IC_{50} literature	IC_{50} [µM] (95%-CI)	IC_{50} literature	IC_{50} [µM] (95%-CI)	IC_{50} literature	IC_{50} [µM] (95%-CI)	IC_{50} literature	IC_{50} [µM] (95%-CI)
**Antidepressants**	Bupropion	161.00 [1]	5.36 [4.19–6.85]	28.60 [1]<br>32.20 [2]	32.86 [21.55–50.10]	738.00 [1]	185.60 [9.36–3681]	115.00 [3]	96.96 [56.01–167.80]
	S-Citalopram		7.15 [5.59–9.15]		59.90 [34.26–104.70]		n.s.		159.80 [83.29–306.50]
	R-Citalopram		5.11 [4.11–6.37]		46.63 [25.56–85.07]		n.s.		58.32 [37.46–90.79]
	Citalopram	13.2 [1]		115.00 [1]		188.00 [1]		116.67 [4]	
**Antiepileptics**	Diazepam	5.0% [5]	n.s.		917.30 [229.70–3663]	2.00 [6]<br>40.00 (mOCT3) [6]	44.46 [36.04–54.85]		29.81 [18.41–48.27]
	Phenobarbital	−6.0% [5]	n.s.		n.s.		n.s.		1027 [39.71–26584]
**Psychostimulants**	*d*-Amphetamine	202 ± 68 [7]<br>96.7 ± 37 [8]	8.39 [6.90–10.20]	10.5 ± 2.6 [7]<br>20.30 ± 16.9 [8]	2.21 [1.70–2.87]	460 ± 140 [7]<br>363 ± 56.4 [8]<br>42 ± 7 [9]<br>41.5 ± 7.5 (DA)<br>24.1 ± 70 (5-HT)[10]<br>n.a [11]	336.6 [179.70–630.40]		71.77 [46.81–110.00]
	Cocaine	85 ± 22 [7]	6.66 [5.28–8.39]	113 ± 21 [7]	27.80 [19.17–40.32]	n.a. [7] [12]	n.s.		n.s.
	α-PVP	1.31 [13]		5.62 [13]<br>17.5 ± 2.5 [14]		1631 [13]<br>>1000 [14]		13.42 [13]	
	R-*α*-PVP		2.15 [1.59–2.90]		13.09 [9.89–17.32]		1016 [364.90–2826]		n.s.
	S-*α*-PVP		1.07 [0.81–1.40]		15.02 [11.28–20.00]		456.7 [144.90–1439]		n.s
**Emerging Therapeutics**	Ketamine	114.5 ± 43.7 [7]<br>73.9 ± 15.2 (K_m) [15]	23.47 [13.34–41.37]	22.7 ± 6.3 [7]<br>33.5 ± 20.3 (K_m) [15]	12.46 [9.71-15.98]	225.7 ± 65 [7]<br>440 [6]<br>52.9 ± 15.0 (K_m) [15]	1028 [589.40–1792]		134.60 [54.70–331.20]
	2-Fluoro-deschloroketamine		14.07 [10.11–19.56]		19.18 [14.89–24.70]		265.5 [167.60–420.50]		n.s.
	MDMA	24.20 ± 9.20 [7]	1.14 [0.90–1.44]	1.63 ± 0.62 [7]	2.71 [2.22–3.31]	73.60 ± 50.30 [7]	153.90 [87.41–270.90]		7.77 [5.92–10.21]
	Psilocin		63.48 [44.97–89.62]		n.s.		n.s.		698.20 [88.81–5488]
**Analgesics**	Tramadol	60.2% [5]<br>30 [16]	5.60 [4.65–6.75]		64.10 [39.58–103.80]		n.s.		74.19 [32.67–168.50]
	O-Desmethyl tramadol	172 [16]	24.16 [19.12–30.54]		371.90 [44.37–3116]		n.s.		n.s.
**Drugs for Narco-lepsy and ADHD**	Methylphenidate		0.36 [0.27–0.46]		28.80 [15.11–54.90]		n.s.		n.s.
	Modafinil		n.s.		n.s.		n.s.		n.s.
Control	Decynium-22		2.66 [2.049–3.44]		4.56 [3.786–5.496]		0.16 [0.1358–0.1904]		0.35 [0.2439–0.5042]

Experiments with MPP$^+$	Experiments with ASP$^+$	% inhbition at 100 µM	Ki (µM) Values	no data available

References

1. Haenisch, B.; Drescher, E.; Thiemer, L.; Xin, H.; Giros, B.; Gautron, S.; Bönisch, H. Interaction of antidepressant and antipsychotic drugs with the human organic cation transporters hOCT1, hOCT2 and hOCT3. *Naunyn-Schmiedeberg's Archives of Pharmacology* **2012**, *385*, 1017–1023. doi:10.1007/s00210-012-0781-8.
2. Sandoval, P.J.; Zorn, K.M.; Clark, A.M.; Ekins, S.; Wright, S.H. Assessment of substrate-dependent ligand interactions at the organic cation transporter OCT2 using six model substrates s. *Molecular Pharmacology* **2018**, *94*, 1057–1068. doi:10.1124/mol.117.111443.

3. Haenisch, B.; Bönisch, H. Interaction of the human plasma membrane monoamine transporter (hPMAT) with antidepressants and antipsychotics. *Naunyn-Schmiedeberg's Archives of Pharmacology* **2010**, *381*, 33–39. doi:10.1007/s00210-009-0479-8.
4. Zhou, M.; Engel, K.; Wang, J. Evidence for significant contribution of a newly identified monoamine transporter (PMAT) to serotonin uptake in the human brain. *Biochemical Pharmacology* **2007**, *73*, 147–154. doi:10.1016/j.bcp.2006.09.008.
5. Ahlin, G.; Karlsson, J.; Pedersen, J.M.; Gustavsson, L.; Larsson, R.; Matsson, P.; Norinder, U.; Bergström, C.A.; Artursson, P. Structural requirements for drug inhibition of the liver specific human organic cation transport protein 1. *Journal of Medicinal Chemistry* **2008**, *51*, 5932–5942. doi:10.1021/jm8003152.
6. Massmann, V.; Edemir, B.; Schlatter, E.; Al-Monajjed, R.; Harrach, S.; Klassen, P.; Holle, S.K.; Sindic, A.; Dobrivojevic, M.; Pavenstädt, H.; Ciarimboli, G. The organic cation transporter 3 (OCT3) as molecular target of psychotropic drugs: Transport characteristics and acute regulation of cloned murine OCT3. *Pflugers Archiv European Journal of Physiology* **2014**, *466*, 517–527. doi:10.1007/s00424-013-1335-8.
7. Amphoux, A.; Vialou, V.; Drescher, E.; Brüss, M.; La Cour, C.M.; Rochat, C.; Millan, M.J.; Giros, B.; Bönisch, H.; Gautron, S. Differential pharmacological in vitro properties of organic cation transporters and regional distribution in rat brain. *Neuropharmacology* **2006**, *50*, 941–952. doi:10.1016/j.neuropharm.2006.01.005.
8. Wagner, D.J.; Sager, J.E.; Duan, H.; Isoherranen, N.; Wang, J. Interaction and transport of methamphetamine and its primary metabolites by organic cation and multidrug and toxin extrusion transporters. *Drug Metabolism and Disposition* **2017**, *45*, 770–778. doi:10.1124/dmd.116.074708.
9. Wu, X.; Kekuda, R.; Huang, W.; Fei, Y.J.; Leibach, F.H.; Chen, J.; Conway, S.J.; Ganapathy, V. Identity of the organic cation transporter OCT3 as the extraneuronal monoamine transporter (uptake2) and evidence for the expression of the transporter in the brain. *Journal of Biological Chemistry* **1998**, *273*, 32776–32786. doi:10.1074/jbc.273.49.32776.
10. Zhu, H.J.; Appel, D.I.; Gründemann, D.; Markowitz, J.S. Interaction of organic cation transporter 3 (SLC22A3) and amphetamine. *Journal of Neurochemistry* **2010**, *114*, 142–149. doi:10.1111/j.1471-4159.2010.06738.x.
11. Mayer, F.P.; Schmid, D.; Owens, W.A.; Gould, G.G.; Apuschkin, M.; Kudlacek, O.; Salzer, I.; Boehm, S.; Chiba, P.; Williams, P.H.; Wu, H.H.; Gether, U.; Koek, W.; Daws, L.C.; Sitte, H.H. An unsuspected role for organic cation transporter 3 in the actions of amphetamine. *Neuropsychopharmacology* **2018**, *43*, 2408–2417. doi:10.1038/s41386-018-0053-5.
12. Mayer, F.P.; Schmid, D.; Holy, M.; Daws, L.C.; Sitte, H.H. "Polytox" synthetic cathinone abuse: A potential role for organic cation transporter 3 in combined cathinone-induced efflux. *Neurochemistry International* **2019**, *123*, 7–12. doi:10.1016/j.neuint.2018.09.008.
13. Maier, J.; Niello, M.; Rudin, D.; Daws, L.C.; Sitte, H.H. The Interaction of Organic Cation Transporters 1-3 and PMAT with Psychoactive Substances. *Handbook of experimental pharmacology* **2021**, *3*, 1–20. doi:10.1007/164_2021_469.
14. Duart-Castells, L.; Nadal-Gratacós, N.; Muralter, M.; Puster, B.; Berzosa, X.; Estrada-Tejedor, R.; Niello, M.; Bhat, S.; Pubill, D.; Camarasa, J.; Sitte, H.H.; Escubedo, E.; López-Arnau, R. Role of amino terminal substitutions in the pharmacological, rewarding and psychostimulant profiles of novel synthetic cathinones. *Neuropharmacology* **2021**, *186*, 1–19. doi:10.1016/j.neuropharm.2021.108475.
15. Keiser, M.; Hasan, M.; Oswald, S. Affinity of Ketamine to Clinically Relevant Transporters. *Molecular Pharmaceutics* **2018**, *15*, 326–331. doi:10.1021/acs.molpharmaceut.7b00627.
16. Tzvetkov, M.V.; Saadatmand, A.R.; Lötsch, J.; Tegeder, I.; Stingl, J.C.; Brockmöller, J. Genetically polymorphic OCT1: Another piece in the puzzle of the variable pharmacokinetics and pharmacodynamics of the opioidergic drug tramadol. *Clinical Pharmacology and Therapeutics* **2011**, *90*, 143–150. doi:10.1038/clpt.2011.56.
